# Transcriptome-wide prediction of heat-sensitive RNA structures in *Zea mays*

**DOI:** 10.3389/fpls.2025.1688991

**Published:** 2025-11-26

**Authors:** Mason W. Eisenhauer, Abdelraouf O. Dapour, Warren B. Rouse, Walter N. Moss

**Affiliations:** 1 Roy J. Carver Department of Biochemistry, Biophysics and Molecular Biology, Iowa State University, Ames, IA, United States; 2 Bioinformatics and Computational Biology Program, Iowa State University, Ames, IA, United States

**Keywords:** heat stress, heat shock factors, covariation, ScanFold, RNA structure, *Zea mays*

## Abstract

**Introduction:**

As global temperatures rise, understanding the potential effects on agriculture and food security is critical. Plant heat stress responses are finely controlled at transcriptional and post-transcriptional levels, with RNA secondary structure now recognized as an important regulator in this process.

**Methods:**

To characterize and identify structure-function relationships in *Zea mays*, we applied the computational approach ScanFold to construct a transcriptome-wide database of RNA secondary structures and associated metrics. This database was analyzed at temperatures ranging from moderate 28°C to extreme 42°C. We performed an in-depth analysis of two heat shock factors, *ZmHsf04* and *ZmHsf17*, as a case study for analyzing thermotolerance in maize.

**Results:**

Using the database, we identified evolutionarily conserved RNA structures across the transcriptome that are sensitive to temperature-induced conformational switching. Our analysis of *ZmHsf04* and *ZmHsf17* yielded two key findings: many predicted structures are supported by significant sequence covariation, indicating evolutionary selection and likely functionality; and several structures of interest also exhibited extreme changes in conformation upon temperature increases.

**Discussion:**

These identified structures in *ZmHsf04* and *ZmHsf17* may regulate gene expression through dynamic changes influencing processes like mRNA maturation, localization, expression, or alternative splicing, providing a rubric for understanding and approaching future studies of the transcriptome-wide dataset. The dataset and methodology presented here provide a rapid and robust approach to facilitate research into plant abiotic stress response, offering a crucial first step in understanding the role of RNA structure in *Z. mays* heat response.

## Introduction

Plant life is critical to the survival of both humans and animals. Although many scientific innovations have improved crop hardiness and annual yield, the impact of rising temperatures and population is becoming a topic of concern. One important area of research that may play a large role in alleviating the problems associated with climate change is understanding plant abiotic stress response (Moustafa, 2016). Abiotic stress is an adverse effect caused by changes in factors such as extreme temperatures, drought, and changes in salinity that impact a plant’s growth and development. These stress factors can have a massive impact on crop production (Zhang et al., 2023) and affect the people who rely on these crops for food. Although plants have evolved mechanisms to sense these stressors and adapt to survive and reproduce, being able to accurately determine changes in response to different stresses is an important step in increasing the quality and quantity of crop yields (Gourdji et al., 2013). One stressor that relates directly to global warming is heat stress, and as such, determining ways to combat the effects of rising temperatures on plants is imperative to maintaining crop yields. One such way that heat stress can affect plants is through changes in gene expression, such as upregulation of heat shock transcription factors (Hsfs) that target heat shock proteins (HSPs) and enzymes that degrade reactive oxygen species (ROS), as well as induce the unfolded protein response (UPR) (Chen et al., 2022). Aside from changes in protein expression, there are also many changes at the RNA level. A classic example of this is through RNA thermometers, which provide a protein-independent, post-transcriptional regulation of gene expression (Raza et al., 2024). When plants experience different temperatures, this can cause RNA secondary structures to adopt different conformations, which lead to variable effects on gene expression (Chung et al., 2020; Thomas et al., 2022). Stressors can also affect the expression of small non-coding RNA, such as microRNAs (miRNAs) and small interfering RNAs (siRNAs), which regulate the expression levels of important proteins (Zuo et al., 2021). Long non-coding RNAs (lncRNAs) have also been shown to be active in many plants, especially in relation to stress response. Additionally, these stressors can affect the levels of RNA modifications, which play a major role in controlling expression (Zhao et al., 2016). The differential expression controlled by RNA and RNA secondary structure is important to consider when trying to understand changes in gene expression caused by abiotic stress.

Although we know that RNA plays an important role in stress response, one of the key aspects regulating its function is the RNA secondary structure (Wan et al., 2011; Assmann et al., 2023). Changes in RNA structure can correlate with alterations in expression in interesting ways: e.g., as with RNA thermometers (Ganser et al., 2019) and heat-induced splicing changes (Deng et al., 2011; Zhang et al., 2020). We now appreciate mRNA as being more than a transient intermediate between DNA and protein, with secondary structures found throughout affecting splicing, localization, and translation (Bullock et al., 2010; Ding et al., 2014; Martin et al., 2023). The untranslated regions (UTRs), especially 3′UTRs, contain the most evidence of functional RNA secondary structures in mRNAs and have been noted as hubs of post-transcriptional regulation (Mauger et al., 2019; Mayr, 2019; Shi et al., 2020). This makes it clear that RNA secondary structures are key regulators of important cellular functions (Shi et al., 2020). Although the importance of these RNA structures is clear, it is still difficult to identify the functionally relevant structures across long sequences, and detailed studies have not been completed for many species (Wan et al., 2011).

To discover structures with a high likelihood of functionality, which may be involved in abiotic stress response, we use our laboratory’s algorithm: ScanFold (Andrews et al., 2018). ScanFold is a functional RNA secondary structure discovery algorithm that deduces local structural stability, propensity for unusual sequence-ordered stability, and likely functionality. This is attained through the generation of consensus structures with base pairs that are weighted by their contribution to the ordered structural stability as measured by the thermodynamic *z*-score (Andrews et al., 2018, 2020). Here, the *z*-score measures the ordered secondary structural stability of predicted structures [using RNAfold (Hofacker, 2003)], as measured by the number of standard deviations either more or less stable than random. Metrics such as the *z*-score and ensemble diversity (ED) are of great importance for predicting functional structures. The ED value is representative of the volatility of the predicted secondary structure, with lower ED values representing a decrease in potential conformations (Su et al., 2019). These conformations may be evolved as a characteristic of some functional RNAs, suggesting selection of particular structures (Moss, 2018). These metrics give insights into the potential functionality of the structures predicted (Clote et al., 2005; Freyhult et al., 2005). Aside from these metrics, the structural data generated from this algorithm can be used in conjunction with programs that analyze conservation and covariation, such as cm-builder (Manfredonia et al., 2020), to add an additional layer of potential functional significance to the predictions (Andrews et al., 2021).

Here, ScanFold is implemented to identify and characterize functional RNA secondary structures of *Zea mays*. Minimal work has been completed to map out the secondary structures in this organism, creating a gap in knowledge for how the RNA structure affects plant gene expression. Using the ScanFold pipeline, we have developed a database for predicted RNA secondary structures and heat-sensitive structures in *Z. mays* (https://zenodo.org/records/17402684). To show how such data could be used, we performed an in-depth analysis of two target genes from the heat shock factor (Hsf) family of genes: *ZmHsf04* and *ZmHsf17*, using generated ScanFold data followed by the Cobretti pipeline. This case study revealed several potentially functional structures with evidence of covariation, including several with evidence of undergoing heat-related changes in structure. These results provide a strong basis for future work to gain insight into the roles of RNA structure in regulating heat stress in maize.

## Materials and methods

### Acquisition of materials

Transcriptome data were accessed from genome assembly Zm-B73-REFERENCE-NAM-5.0 as found on MaizeGDB (Woodhouse et al., 2021). The fasta file containing the entire transcript, including every protein-coding sequence, was downloaded and manually split into individual files for use with ScanFold 2.0 (Andrews et al., 2018, 2022). For ease of viewing within Integrative Genomics Viewer (IGV) (Robinson et al., 2011), the GFF3 file (used to view gene isoforms) and genome-wide fasta (including every protein-coding sequence) files were downloaded and loaded into the program.

### ScanFold

ScanFold 2.0 consists of a scanning step, ScanFold-Scan, and a folding step, ScanFold-Fold. In ScanFold-Scan, a 120-nucleotide (nt) scanning window is used to analyze the sequence of interest (here, the *ZmHsf04* and *ZmHsf17* genes from the Zm-B73-REFERENCE-NAM-5.0 genome on MaizeGDB). RNAfold (Woodhouse et al., 2021; Andrews et al., 2022) is used on the sequence in each window to calculate its native minimum free energy (MFE) and associated secondary structure. Using mononucleotide shuffling, the native sequence within each window is shuffled and folded 100 times to calculate an average randomized MFE value. These MFE values are then used to calculate the thermodynamic *z*-score. Using all *z*-scores, ScanFold-Fold generates a consensus secondary (centroid) structure model. This model is based on paired nucleotides that recur across low *z*-score analysis windows (Ding et al., 2005). The resulting structures are biased towards sequence-ordered stability (low *z*-scores), which suggests a likely role of evolution in preserving the sequence order for a functional purpose. Structures with low *z*-scores are then extracted to undergo further analysis. Metrics obtained from ScanFold include MFE, Δ*G*, *z*-score, ED, and a centroid structure (a secondary structure representing the tendencies of the greater ensemble of potential structures) (Ding et al., 2005; Freyhult et al., 2005). In addition to these important metrics, several loadable wig and bp files for depicting the predicted base pairs are generated, allowing the data to be visualized through a genomics viewer such as IGV (Robinson et al., 2011; Thorvaldsdóttir et al., 2013).

### Initiating ScanFold runs on each gene of *Zea mays*

Each fasta file containing a protein-coding sequence, numbering 39,755 sequences in total, was uploaded to the Iowa State High Performance Computing cluster (Nova) and used as input in ScanFold 2.0 (Andrews et al., 2018, 2022). ScanFold default settings were used with a window size of 120 nt, a step size of 1 nt, randomizations of 100, and mononucleotide shuffling. The temperature used to create the database of full runs was 37°C. Using default parameters allows for the algorithm to be run using the following generic command: python ScanFold2.py Zm00001eb049300.fasta –folder_name Zm00001eb049300_chr1. Subsequent simplified ScanFold-Scan runs, which removed the refolding step and *z*-score calculation, used two temperatures: 28°C and 42°C. Running ScanFold in this way gives us multiple outputs of each gene, at the chosen temperatures of 28°C, 37°C, and 42°C, allowing for analysis of heat stress effects on these genes. Outputs from this step totaled approximately 255 GB of data, all made publicly available (https://zenodo.org/records/17402684).

### Covariation studies and analysis

Covariation studies, analyzing sequences for covarying nucleotide pairs that may indicate an evolutionary conservation of particular structures, were completed using the Cobretti pipeline (Peterson et al., 2023). This pipeline was initiated on these genes to aid in the identification of covarying pairs and potential pseudoknots. Using Biopython 1.70 and Python 3.6.5 on the Iowa State High Performance Computing cluster, each stage of the Cobretti pipeline was run as follows, changing the stage as necessary: python cobretti.py -stage 1AB. Substage 1AA was completed manually in the previous step, leaving substage 1AB for the BLAST protocol, which ran using default settings. Once completed, stage 1B was initiated, where cm-builder was run. The cm-builder pipeline generated homologous alignments of motif sequences against an NCBI BLAST database using the Infernal (Inference of RNA Alignment) program (Nawrocki and Eddy, 2013; Peterson et al., 2023). Cobretti trims motifs into nested substructures where unpaired nucleotides from the 5′ and 3′ ends are removed to reduce noise (Tavares et al., 2019), allowing for Infernal to analyze them. With the completion of the cm-builder substage, stage 1C was completed to run R-Scape (RNA Structural Covariation Above Phylogenetic Expectation) (Rivas et al., 2016) and to clean up data. R-Scape analysis facilitates finding structures of interest based on the number of covarying pairs and their associated statistical power, which helps to establish how robust the constraint of structure may be on sequence evolution. Through this process, significant covariation may be determined (Rivas, 2021). R-Scape was run using default settings, where alignments with high powers (>0.10) and that met the *E*-value threshold of 0.05 were considered significant (Tavares et al., 2019). From there, predicted pseudoknots or variable structures are gathered, as well as checking stockholm files to verify the integrity of predicted data.

### Analysis of changes in ensemble diversity between temperatures

Data acquired from running ScanFold 2.0 (Andrews et al., 2022) are extracted and compared to data from other temperatures, with emphasis on discovering the change in ensemble diversity (ΔED) between the 28°C and 42°C datasets. We employed a statistical approach, where the ΔED was averaged across all analysis windows for each gene and compared to global database values. A one-sample *t*-test was conducted on the Hsf family of genes to determine significance in the differences in the data. The average ΔED across the dataset and standard deviations for both the database and Hsf family were calculated, allowing for the calculation of a statistical standard score for each gene. The largest changes in ED, positive or negative, in Hsf genes were selected for further analysis, with some structures being chosen based on previously published information. From here, centroid structures are extracted and modeled for each temperature to compare the similarities and/or differences, as a potential structure–functionality relationship may be predicted from these changes.

### Visualization of data

Using the Zm-B73-REFERENCE-NAM-5.0 assembly, as well as the GFF3 files and ScanFold output files, data from each gene can be visualized when imported into IGV. Files from ScanFold will need to be converted to correct orientation and coordinates, which is done using created scripts (https://github.com/moss-lab/Maize_Project_Scripts/tree/main) based on coordinates and strand directionality pulled from MaizeGDB (Woodhouse et al., 2021). These scripts include DeltaED_Calculator, which generates a DeltaED wig file compatible with IGV, and IGV_File_Preparation, which scans the ScanFold output and creates files compatible with IGV based on the coordinates provided in the fasta file. This allows for accessible visualization of metrics in comparison to predicted base pairings and regionality within the gene. Visualization of structural motifs can be done using VARNA (Darty et al., 2009) by loading the nucleotide sequence and dot bracket notation of a particular structural motif as extracted from ScanFold data.

## Results

### Interpreting and using the database

We present a comprehensive transcriptome-wide database of RNA secondary structures and associated metrics for *Z. mays*, derived from the analysis of 39,755 transcripts using the ScanFold pipeline (https://zenodo.org/records/17402684). This dataset, totaling approximately 255 GB, has been highly compressed and optimized to a final size of only 33 GB, allowing researchers to easily download it as a single dataset. In addition, we provide multiple access options so users interested in only a single gene—or a subset of genes—can retrieve the data directly without needing to unzip the full dataset. Each entry is named according to the Ensembl Plants nomenclature (e.g., Zm00001eb049300), reflecting gene identifiers used in the *Z. mays* B73 reference genome (Zm-B73-REFERENCE-NAM-5.0).

The dataset is structured hierarchically within gene-specific folders, each containing the following components: a FASTA file (“.fasta”) storing the transcript sequence for the corresponding gene and a subfolder named with the gene ID and chromosome suffix (e.g., “Zm00001eb049300_chr1”). This subfolder includes a “win_120_step_1.tsv” file with ScanFold scan output per window, an “igv_files” folder with tracks for visualization in the IGV, and an “extracted_structures” folder housing all predicted motifs and substructures for the transcript. Additionally, a folder mirroring the parent folder name contains the final ScanFold results: extracted sub-motifs in “.txt” format, consensus structures in dot-bracket (“.dbn”), and CT (“.ct”) formats for no *z*-score and *z*-score thresholds of −1 and −2, and intermediate output files generated during ScanFold processing. This clear and logical structure ensures that users can navigate the dataset with ease, accessing detailed structural data for individual transcripts or the full dataset.

To enhance accessibility and accommodate users across different operating systems (Linux, macOS, and Windows), we provide multiple recommended methods—as described in **Supplementary File S1**—to access the Maize ScanFold dataset. These options allow users to either download the dataset in full or selectively retrieve a single gene of interest while preserving its hierarchical structure, including all associated subfolders and files, without the need to manually decompress or navigate the entire archive. The dataset is organized into clearly labeled directory paths, enabling efficient extraction or copying of individual genes using standard tools such as pigz and tar for high-speed streaming extraction, ratarmount for mounting the archive as a virtual read-only filesystem, or 7-Zip for Windows users. These approaches ensure maximum flexibility, allowing researchers to access only the data they need with minimal storage requirements and effort.

### Statistical analysis and comparison of heat shock factors

**Figure 1:**
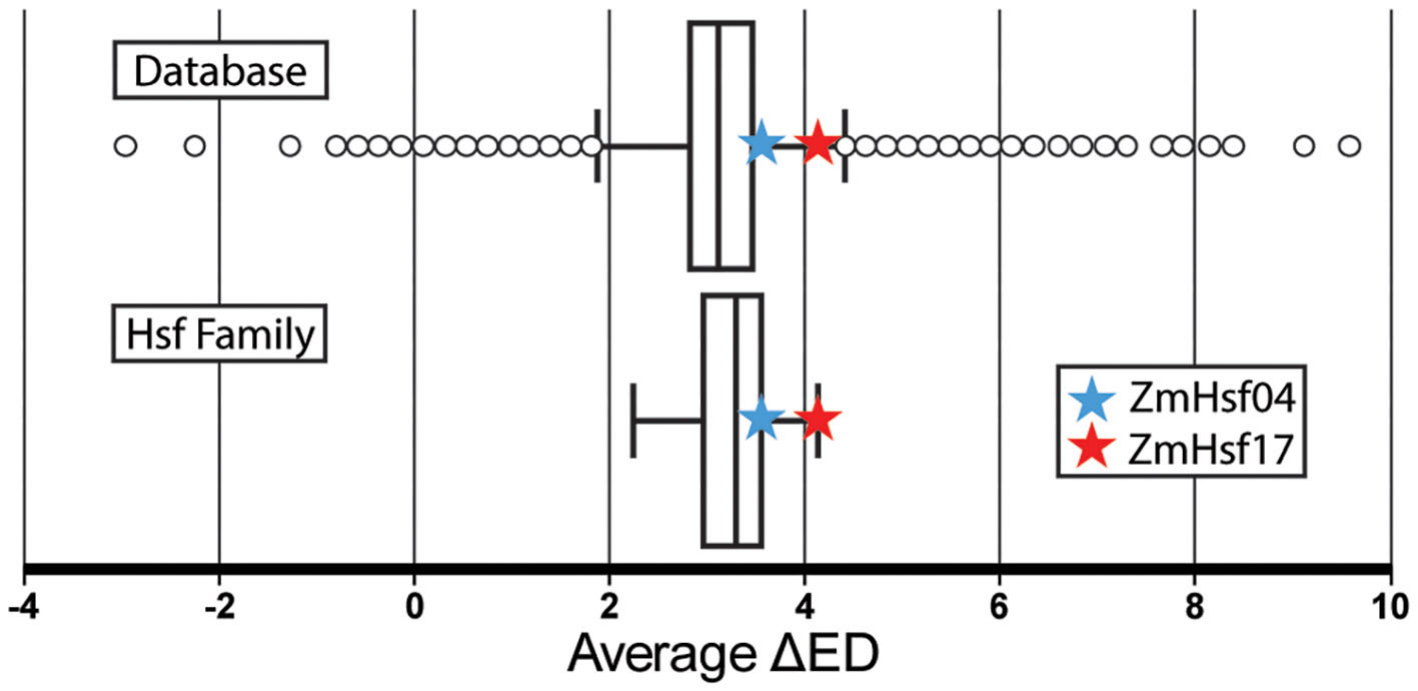
Box plot of ScanFold-Scan per gene average ΔED data (unitless) collected between 28°C and 42°C for the entire database and the heat shock factor (Hsf) family of genes in *Z. mays*. *ZmHsf04* and *ZmHsf17* genes are denoted with blue and red stars, respectively. *ZmHsf17* is shown as the largest average ΔED in the Hsf family, and *ZmHsf04* is in the third quartile of the Hsf family.

With interest in discovering structures related to changing temperatures, we focused on the Hsfs in maize as a case study due to their known expressional differences under elevated temperatures (Jiang et al., 2021; Li and Howell, 2021; Fan et al., 2025). From the transcriptome-wide database, aggregated data of the average changes in ED (ΔED) between 28°C and 42°C were collected and compared. Notably, the average for each window in each gene had a value of 3.16, suggesting an overall higher ED at increased temperatures, as expected. The median ED value sat at 3.12, with first and third quartile values being 2.83 and 3.46, respectively. Thus, a tight increase of approximately 3 points average ΔED is to be expected for this dataset. Determining values that either stray from this observation or sit on the higher end of values may suggest potential functionality due to the temperature. To determine key differences between the Hsf genes and a larger database, the gene IDs and respective average ΔED values were compiled to allow for comparison (**Supplementary Table S1**). As such, the targeted Hsf family’s individual average ΔEDs were pulled and compared to the aggregate data (**Figure 1**).

**Figure 2:**
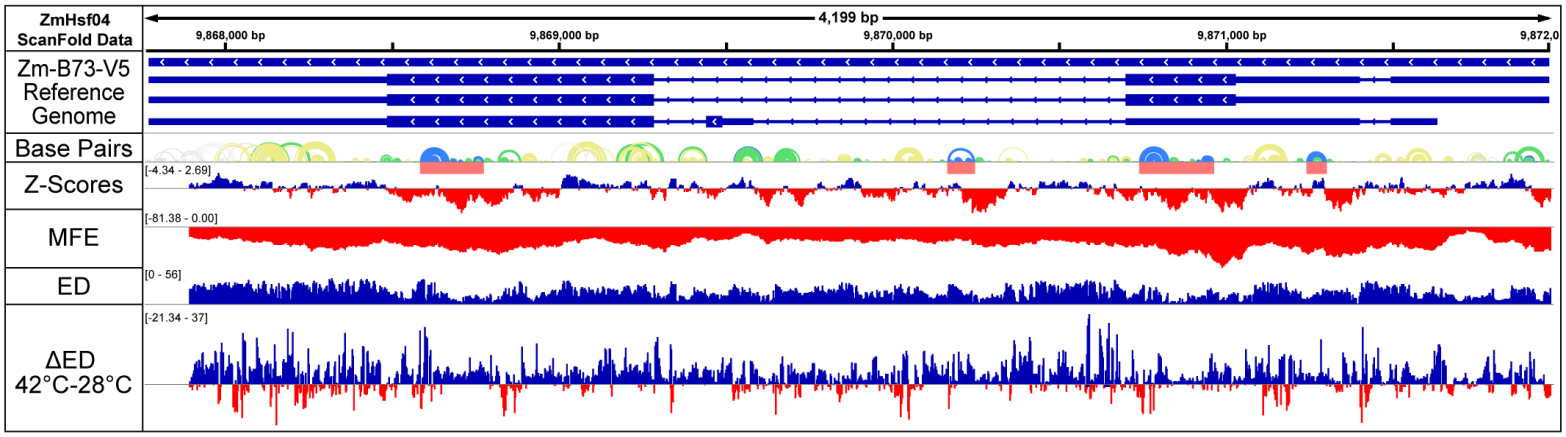
ScanFold data for the *ZmHsf04* (Zm00001eb301280) gene at 28°C. Gene cartoons represent all annotated isoforms found on chromosome 7 of the *Z. mays B73-V5* genome. ScanFold predicted base pairs are represented as structures with *z*-scores ≥0 shown in gray, <0 shown in yellow, ≤−1 shown in green, and ≤−2 shown in blue, respectively. Four of the prominent structures with *z*-scores ≤ −2 are designated with a red bar. ScanFold per window *z*-scores, minimum free energies (MFEs), and ensemble diversity (ED) metrics show the ordered stability, thermodynamic stability, and conformational volatility of *ZmHsf04*. The change in ED (ΔED) shows the regions that are most sensitive to ensemble structure change, going from moderate (28°C) to high temperature (42°C). Positive ΔED values suggest a larger ensemble of potential structures in the window at 42°C while negative ΔED values suggest a smaller ensemble of potential structures in the window at 42°C.

The Hsf family of genes is not significantly different in ΔED values when compared to the aggregate database, although a slight average increase is seen. A one-sample *t*-test was conducted, in which a standard error value of 0.73 and a *p*-value of 0.47 were calculated, failing to reject the null hypothesis and confirming this insignificant difference in ΔED values. Of the Hsf genes, *ZmHsf17* has the largest increase in ΔED at 4.13 points, giving it a standard score of 2.01 against Hsf genes and 1.52 against the entire transcriptome. Previous work on *ZmHsf17* has found it to be involved in the positive regulation of *ZmPAH1*, increasing thermotolerance in maize, further showing the importance of this gene (Zhang et al., 2025). Also of note is *ZmHsf04*, with a much greater representation of −2 (or lower) *z*-score structures, totaling 11 motifs, than other Hsf genes (**Figure 2**). It has been demonstrated that *ZmHsf20* negatively regulates *ZmHsf04*, wherein by overexpressing *ZmHsf04*, improved heat tolerance in maize seedlings was achieved (Li et al., 2024b). Although the average change in ED across this gene was not extremely high, local regions of large ΔED were clearly present in locations of interest, including UTRs and intronic regions. Thus, based on extreme average ΔED (*ZmHsf17*) and the presence of thermodynamically stable and ordered sequences (*ZmHsf04*), we selected these two Hsf genes for additional analyses.

### Heat shock transcription factor *ZmHsf04*

The *ZmHsf04* gene, located in chromosome 7, encodes an Hsf found in *Z. mays*, which has been shown to improve thermotolerance in transgenic *Arabidopsis* (Jiang et al., 2018). This gene has three potential splice variants, two of which affect the 5′UTR and one that affects both the 5′UTR and the coding DNA sequence. ScanFold predicts that this gene is relatively thermodynamically stable with regions of higher stability in the 5′UTR, exhibiting a peak MFE of −81.38 kcal/mol, and a large intron, exhibiting a peak MFE of −51.51 kcal/mol. The *z*-scores fluctuate significantly across the gene with large portions of the UTRs, exons, and introns displaying positive values (i.e., regions evolved to be unstructured), as well as negative values (i.e., regions evolved to be structured). A total of 11 structural motifs with a *z*-score of −2 or lower were identified across all regions of the *ZmHsf04* gene (**Figure 2**). Interestingly, the 3′UTR is devoid of −2 *z*-score structures, whereas the exons appear to be slightly enriched. Additionally, many structures with a *z*-score between −1 and −2 were also present throughout the gene, with several spanning intron–exon junctions, UTR splice sites, and the stop codon. Aside from low *z*-score structures, many regions undergo a significant change in ensemble diversity (ΔED) when ScanFold is run at different temperatures. Of these, a particular window near the beginning of the large intron sees a ΔED of 37.38 points, suggesting a potential loss of a structured region. These regions of significant ΔED, positive or negative, offer insight into structures that may be dynamic and adopt different conformations depending on the conditions.

**Figure 3:**
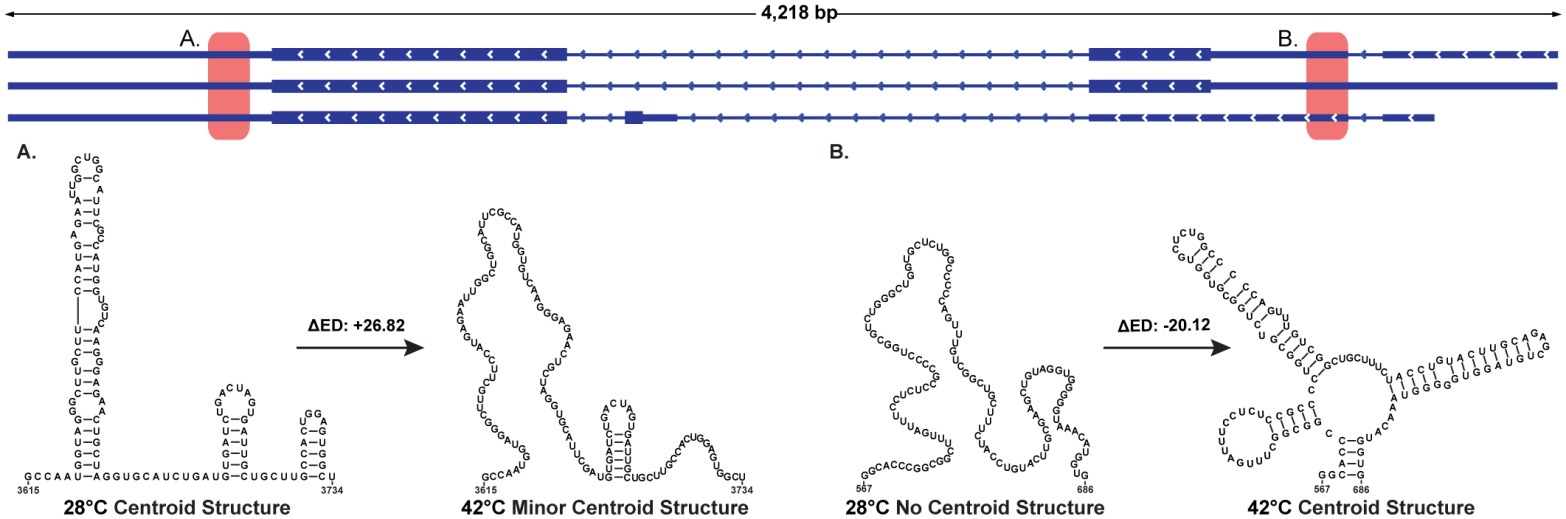
Select centroid structures from *ZmHsf04* at 42°C and 28°C. These structures were chosen through comparison of windows with the largest change in ensemble diversity (ΔED) between 42°C and 28°C. Here, a +ΔED indicates an increase in ED with temperature, and a −ΔED indicates a decrease in ED with increased temperature. **(A)** This window in the 3′UTR has a +ΔED and a loss of centroid structure with increased temperatures. **(B)** This window at the 5′UTR–intron junction has a −ΔED and a gain of centroid structure with increased temperatures.

Of particular interest are two regions in *ZmHsf04* (**Figure 3**) with large changes in ED. These two regions are found in the 5′ and 3′UTR, which are known hubs of mRNA regulation (Gu et al., 2014; West et al., 2025). Here, the effect of temperature increases on the ED can be visualized by comparing the centroid structure (the structure that best represents the tendency of the structural ensemble) at each temperature. The region in the 3′UTR (**Figure 3A**) was discovered to have a less structured centroid and a higher ED, gaining 26.82 points, at 42°C compared to 28°C. In contrast, the region at the 5′UTR–intron junction was discovered to have a more structured centroid and lower ED, losing 20.12 points, at 42°C compared to 28°C (**Figure 3B**). Based on the location of these regions, the changes in structure could play a role in post-transcriptional regulation. Of potential significance is the known alternative splicing that has been noted at this splicing junction (Zhang et al., 2020), which may suggest a functional role for this conformationally dynamic RNA secondary structure.

**Figure 4:**
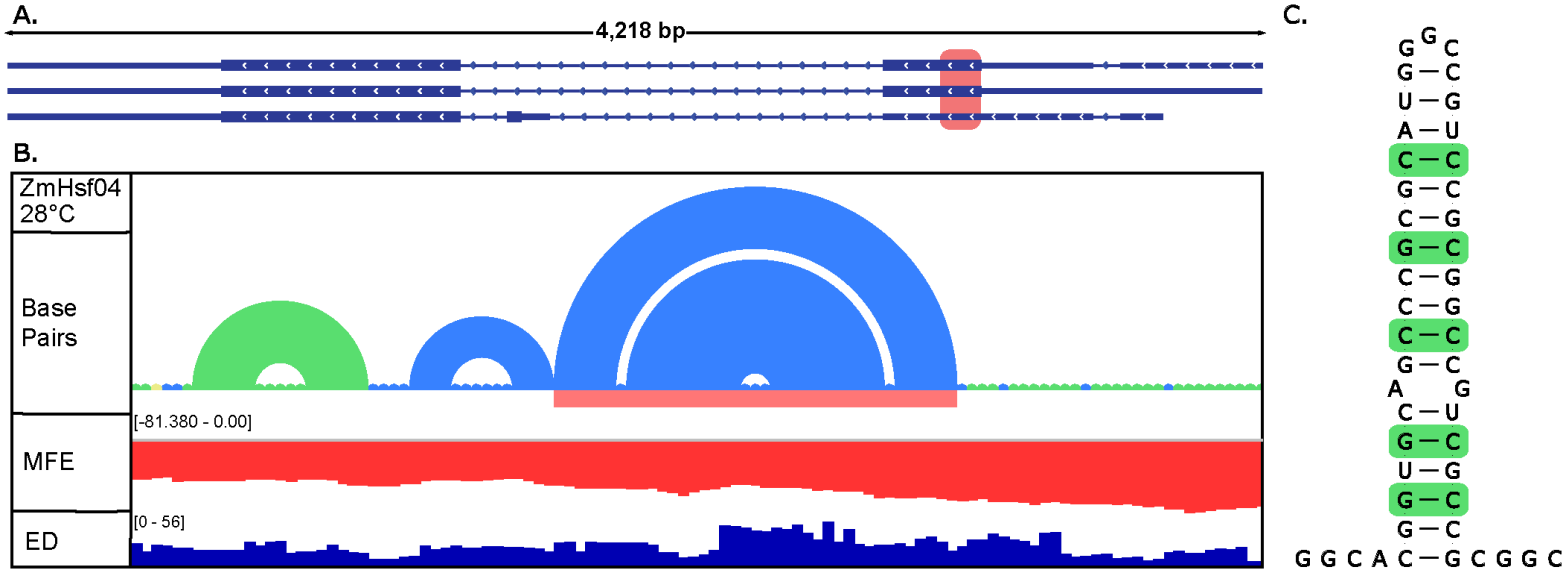
Local ScanFold metrics and structure showing covarying pairs in the 5′UTR of *ZmHsf04*. **(A)** Isoforms for *ZmHsf04* with the local region of interest highlighted in light red. **(B)** Local metrics of the region are highlighted in **(A)** showing base pairs where −1 and −2 *z*-scores are shown as green and blue, respectively, *z*-scores, minimum free energy (MFE), and ensemble diversity (ED). The light red region under the base pairs shows the extracted structure displaying covariation. **(C)** The extracted hairpin structure showing five covarying pairs, as found through the cm-builder portion of Cobretti, highlighted in green.

From further analysis of changes in centroid structure and *z*-score, and from analysis of covariation of the *ZmHsf04 z*-score structures ≤ −2, another region of interest was discovered that suggests conservation of base pairings and structure (**Figure 4**). Through covariation analysis, support of functionality may be strengthened due to the likelihood of regions conserving structure (Peterson et al., 2023). Depending on the isoform considered, this discovered structure fell in either exon 1 or the 5′UTR region of *ZmHsf04* (**Figures 4A, B**). Upon deeper analysis, one structure displaying a hairpin, had evidence of five statistically significant covarying base pairs (**Figure 4C**; **Supplementary Figure S1**), one of which displayed a power of 0.88. Interestingly, this structure had significant GC nucleotide content as well, which is known to affect the structures and stability of regions (Zhang et al., 2011; Cornwell-Arquitt et al., 2025). Of the windows containing this structure, the lowest predicted *z*-score reached −3.79, while most windows settled near −3. Notably, being a motif with a *z*-score below −2 (**Figure 4B**) and possessing numerous covarying pairs supports a higher likelihood of functionality.

### Heat shock transcription factor *ZmHsf17*

**Figure 5:**
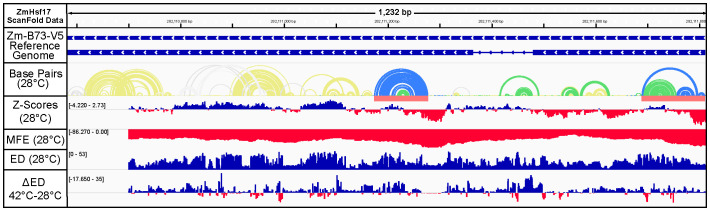
ScanFold data for the *ZmHsf17* (Zm00001eb056980) gene at 28°C. Gene cartoons represent all annotated isoforms found on chromosome 1 of the *Z. mays B73-V5* genome. ScanFold predicted base pairs are represented as structures with *z*-scores ≥ 0 shown in gray, < 0 shown in yellow, ≤ −1 shown in green, and ≤ −2 shown in blue, respectively. Prominent structures with *z*-scores ≤ −2 are designated with a red bar below the base pair arcs. ScanFold per window *z*-scores, minimum free energies (MFEs), and ensemble diversity (ED) metrics show the ordered stability, thermodynamic stability, and conformational volatility of *ZmHsf17*. The change in ED (ΔED) shows the regions that are most sensitive to ensemble structure change, going from moderate (28°C) to high temperature (42°C). Positive ΔED values suggest a larger ensemble of potential structures in the window at 42°C while negative ΔED values suggest a smaller ensemble of potential structures in the window at 42°C.

*ZmHsf17*, located in the first chromosome of *Z. mays*, has one annotated isoform and no annotated UTRs. ScanFold identified three motifs with *z*-scores of −2 or less and 3 motifs with *z*-scores between −1 and −2. All the −2 structures are in the exonic regions; however, one of the −1 structures is located at an intron–exon junction (**Figure 5**). Interestingly, all −2 structures are found near the beginning of their respective exons. The *z*-scores fluctuate significantly across the second exon, with positive *z*-scores being very prominent after the initial stretch of negative *z*-scores, suggesting evolution towards an unstructured region. *ZmHsf17* was also found to be relatively thermodynamically stable with one region associated with a −2 *z*-score structure in exon 2, predicting an MFE of −86.27 kcal/mol. Additionally, the ≤−1 *z*-score structure spanning the exon 1–intron 1 junction is shown to have the largest change in ED between 28°C and 42°C. This intron undergoes alternative splicing, making it a region of high interest (Zhang et al., 2020). The largest changes in ED across the gene tend to be near intron 1 or within exon 2, while exon 1 appears mostly unaffected, except near the exon 1–intron 1 junction as previously noted.

**Figure 6:**
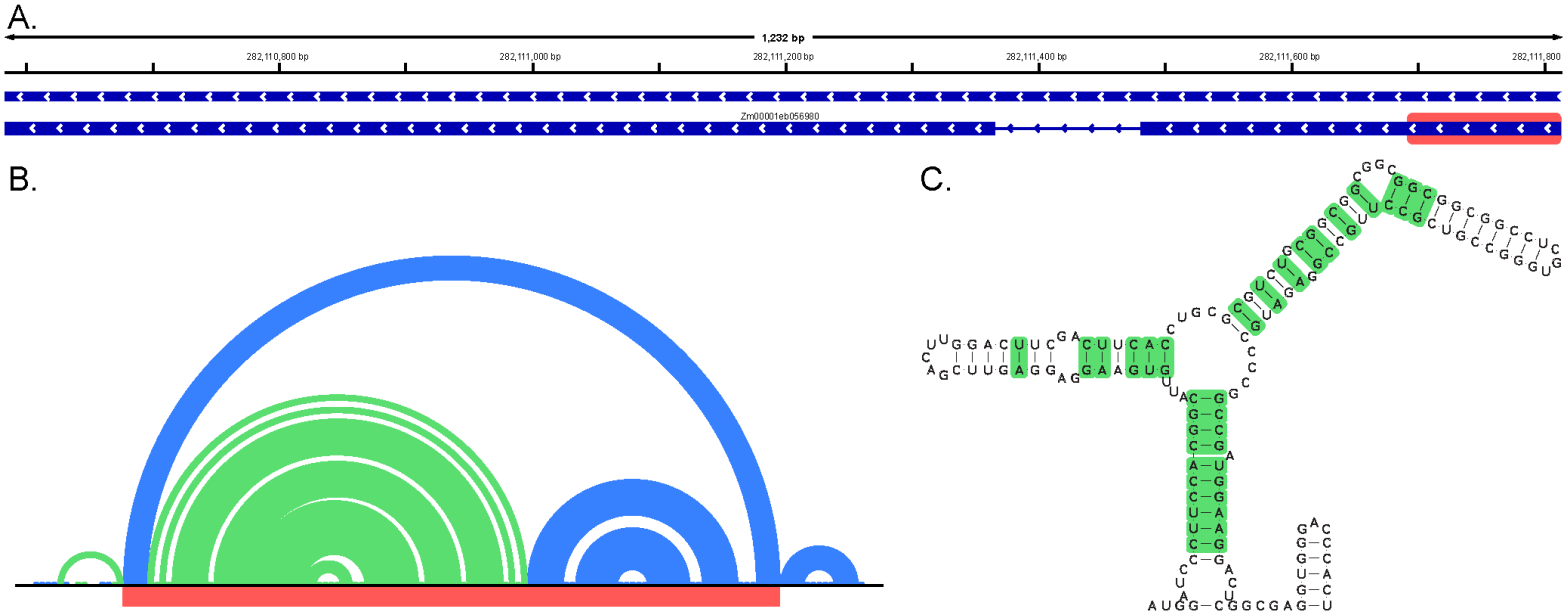
Local region data in exon 1 of *ZmHsf17* showing a covarying structural motif in its different predicted forms. **(A)** Isoforms for *ZmHsf17* with the local region of interest highlighted in light red. **(B)** Local ScanFold predicted base pairing of the region where −1 and −2 *z*-score structures are shown with green and blue, respectively. **(C)** HFoldScanFold predicted structure from Cobretti with 26 covarying pairs shown highlighted in green.

The −2 *z*-score structure near the beginning of exon 1, with windows suggesting a *z*-score as low as −4.22, was found to have high levels of covariation (**Figure 6**). Interestingly, when this region is folded using ScanFold (**Supplementary Figure S2**) or different pseudoknot prediction tools (**Figure 6C**; **Supplementary Figure S2**), there are small changes seen, but the structure always maintains high levels of covariation. Maintenance of covariation suggests that this structure may be dynamic, and the variations in structure may all be biologically relevant. Notably, this covariation is seen across homologous heat stress transcription factors in *Setaria italica*, *Prunus dulcis*, *Glycine max*, and numerous other species, as can be seen in the alignment (**Supplementary Figure S3**). These factors all suggest a potential structure–function relationship located within this region.

**Figure 7:**
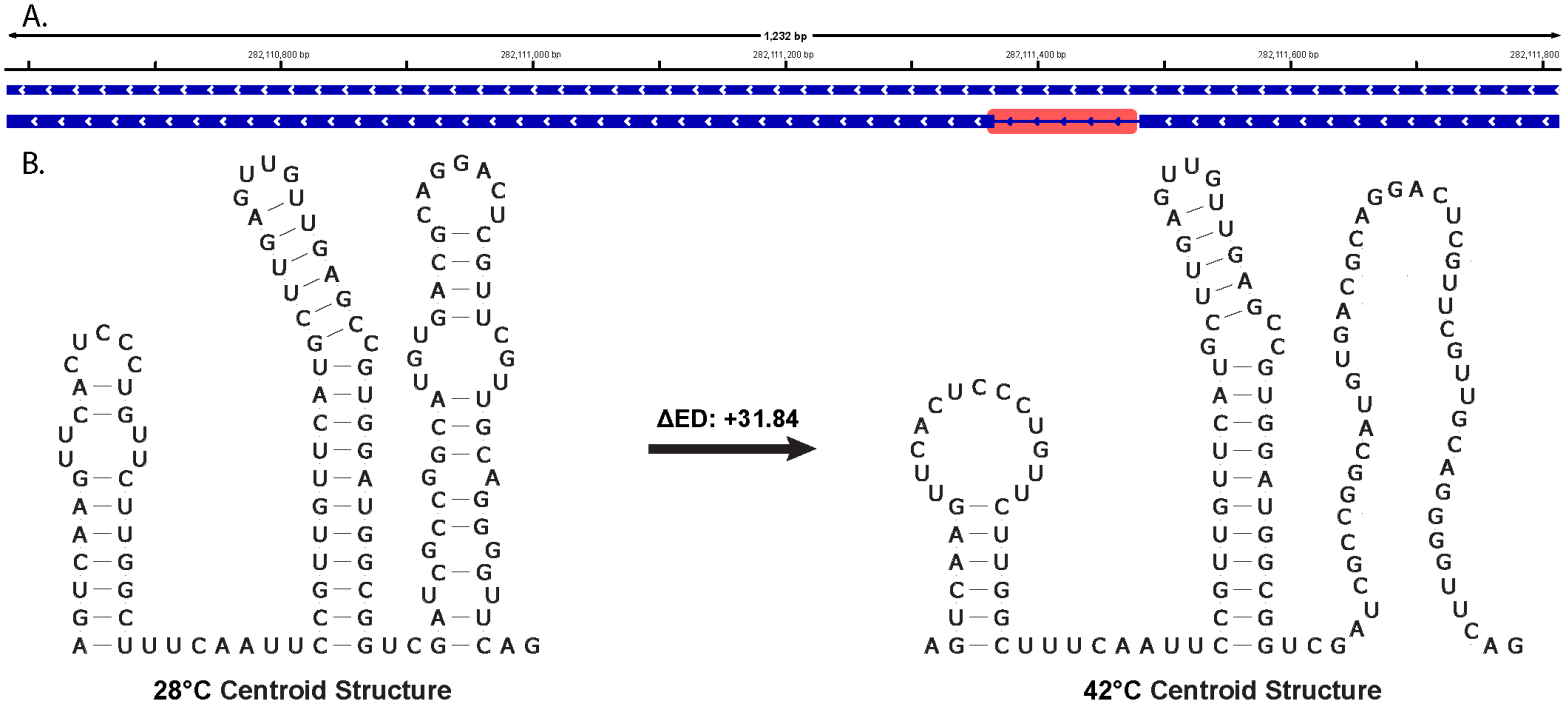
Local region of interest in the intron of *ZmHsf17* showing a region of large change in ensemble diversity (ΔED) between 42°C and 28°C. Here, a +ΔED indicates an increase in ED with temperature. **(A)** Isoforms of *ZmHsf17* with the region of interest highlighted in red. **(B)** Predicted centroid structural change between temperatures in this region that may influence gene expression.

Like *ZmHsf04*, the first intron of *ZmHsf17* also undergoes alternative splicing (Zhang et al., 2020). Although with an insignificant *z*-score, settling at −0.48, the ΔED and the centroid structure of a region spanning the exon 1–intron 1 junction, suggesting a potential temperature-induced change, were discovered (**Figure 7**). Many consecutive windows have large ΔED values, the most significant of which is at +31.84 points (**Figure 7B**), suggesting that this region may sample more conformations at higher temperatures. When looking at the centroids predicted under both temperatures, the higher temperature causes a decrease in the structuredness of the region, particularly in one of the associated hairpins (**Figure 7C**). The loss of an entire hairpin and the slight rearrangement of others could alter any associated function, specifically if these changes are affecting the location and accessibility of splicing regulatory elements.

## Discussion

In plants, RNA plays an important role in stress response, with RNA secondary structures being a key aspect regulating its function (Wan et al., 2011). Changes in RNA structure can lead to altered gene expression through various mechanisms, including alternative splicing (Deng et al., 2011; Ganser et al., 2019; Zhang et al., 2020). This study, using *Z. mays* as a model, provides a crucial initial step by identifying numerous previously uncharacterized and potentially functional RNA structures. Through in-depth analysis of *ZmHsf04* and *ZmHsf17*, we demonstrate the utility of our created database, while also noting the presence of interesting, highly ordered RNA structures within these genes. These identified structures likely serve diverse regulatory purposes depending on their specific gene context and location, laying important groundwork for understanding their roles in plant abiotic stress response.

Our newly developed database provides full ScanFold runs for *Z. mays* at 37°C, alongside ScanFold-Scan data illustrating changes in various metrics between 28°C and 42°C. While these predicted structures largely exhibit robustness, with only a few regions showing extreme changes, further experimental validation across a wider range of temperatures is crucial to fully elucidate their dynamic structure–function relationships. The comprehensive nature of this dataset, encompassing average ΔED values across the entire transcriptome and specific gene families like the Hsf family of genes, enables the identification of promising targets based on their thermodynamic properties and their predicted responsiveness to temperature fluctuations. This methodology is highly adaptable; for example, researchers can leverage this database to predict the potential role of RNA secondary structures in heat shock response for previously identified genes of interest. Furthermore, the approach presented can be broadly applied to compare data across entire gene families or even the whole genome, as exemplified in **Figure 1**, providing a powerful tool for initial screening and hypothesis generation. This framework is not limited to heat stress and could potentially be extended to explore RNA structural dynamics under other abiotic stresses, such as salinity or drought, through similar computational analyses followed by targeted experimental validation.

While ΔED provides a valuable initial screening metric, it is important to acknowledge its limitations in identifying all functionally relevant structures. Additional statistical analyses, such as examining average *z*-scores or changes in *z*-scores, can complement ΔED to more comprehensively identify genes likely to possess functional RNA secondary structures. This multi-metric approach is important because relying solely on gene-wide average ΔED might overlook localized regions of significant structural change or highly stable, functional structures that do not exhibit a large overall gene-wide ΔED. For instance, despite not showing a significant average ΔED across the entire gene, *ZmHsf04* was selected for further in-depth analysis due to the presence of thermodynamically stable and ordered sequences (low *z*-scores) and its association with previously identified alternative splicing sites (Zhang et al., 2020).

Structural alterations in RNA, whether due to heat stress or other environmental factors, influence post-transcriptional regulation. For instance, proteins often rely on specific RNA secondary structures or modifications to recognize and bind their target transcripts; alterations in these motifs can prevent protein binding, thereby disrupting normal gene expression or function (Vandelli et al., 2024). Structural elements also play a role in mRNA maturation, where RNA splicing, and especially alternative splicing, is a common mechanism to change expression. Furthermore, structures within the 3′UTRs of mRNAs in wheat display a strong correlation with mRNA stability (Wu et al., 2024), and structure profiling in rice identified the role of RNA structure in RNA degradation when tested at a 42°C heat shock (Zhang and Ding, 2021). The computational predictions presented here, particularly those showing a change in predicted RNA structures, may aid in discovering the precise molecular mechanisms underlying these expressional changes.

As illustrated in **Figures 3**, **7**, our analysis reveals that the intronic regions of *ZmHsf04* and *ZmHsf17* harbor predicted structures with potential functional significance, as indicated by their *z*-scores. Despite often being overlooked, introns are rich in cis-regulatory sequence elements important for splicing machinery recognition and overall organismal survival. RNA secondary structure plays diverse roles in both maintaining normal splicing and modulating alternative splicing (Li et al., 2024a). An important consideration arising from our findings is the potential influence of these predicted intronic structures on known alternative splicing events within these genes (Zhang et al., 2020). Gene expression analysis in *ZmHsf04-*expressing transgenic *Arabidopsis* plants, previously completed at a 37°C heat shock against a control, showed that associated HSPs were upregulated (Jiang et al., 2018). This experimental upregulation, alongside known alternative splicing events for the gene, suggests that our identified structures with covarying base pairings may reflect true functional elements. Overall, the observed temperature-induced changes in ED and centroid structures strongly suggest that dynamic alterations in these intronic RNA structures could play roles in plant adaptation to heat stress.

Beyond intronic regions, our findings also highlight RNA structures present within the UTRs of *ZmHsf04* and *ZmHsf17* (**Figures 2**, **5**). The 5′ and 3′UTRs are universally recognized as crucial regulatory hubs in gene expression across all organisms (Mauger et al., 2019; Mayr, 2019; Shi et al., 2020). The identification of thermodynamically stable structures in these regions, combined with their inherent propensity for regulatory function, strongly suggests that these UTR structures play a significant role in modulating the expression of *ZmHsf04* and *ZmHsf17*. Particularly compelling is a motif identified near the beginning of *ZmHsf17*, which exhibits substantial covariation (**Figure 6**). Alignments from the Cobretti pipeline may be reviewed as well to see conservation of such structures and the organisms in which it is seen (**Supplementary Figures S1**, **S2**). Such structures, with robust computational support for their stability and potential evolutionary conservation, represent exceptional candidates for in-depth functional characterization.

Acknowledging the computational nature of this study, a notable limitation is the current absence of experimental validation for these predicted structures, their temperature-induced rearrangements, and their precise functional roles. It should be noted that while these computational studies may often provide promising insights into the functional roles of predicted RNA structures, false readings and predictions are possible. While our analysis of conservation and covariation in *ZmHsf04* and *ZmHsf17* lends to an increased confidence in these structural predictions, extensive further experimental work is warranted. Future studies should include biochemical structure probing, gene expression assays, and detailed analysis of temperature-associated splicing changes to confirm and elucidate the functional significance of these findings. Nevertheless, this study stands as a foundational step, providing a transcriptome-wide database and methodology that significantly advance our understanding of the likely important roles played by RNA secondary structure in abiotic stress response in *Z. mays*.

## Conclusion

*Z. mays* plays a significant role in global energy and food systems. Given the increasing concerns about climate change and rising global temperatures, understanding how RNA secondary structure influences variable gene expression is essential for improving crop yields and ensuring food security. The dynamic RNA structural changes identified in this study offer insights that can inform future genetic engineering strategies aimed at enhancing crop resilience to various abiotic stressors. While acknowledging the necessity of further experimental validation, this study provides a robust transcriptome-wide database and methodology for identifying potentially functional RNA structures in *Z. mays* and predicting their alterations under heat stress conditions.

## Data Availability

The datasets presented in this study can be found in online repositories. The names of the repository/repositories and accession number(s) can be found in the article/**Supplementary Material**.
