# Supplementary material for: Transcriptome-wide prediction of heat-sensitive RNA structures in Zea mays
Source: Front Plant Sci. 2025 Nov 26;16:1688991. doi: 10.3389/fpls.2025.1688991 (PMC12689509; doi:10.3389/fpls.2025.1688991)
Supplement: Supplementary file 1 [file DataSheet1.pdf]

Supplementary File S1: Guide to Accessing the Maize ScanFold Dataset

This document provides detailed instructions for downloading and accessing the data within the Maize_ScanFold_Data_Archive.tar.gz archive. The dataset is a comprehensive collection of ScanFold analysis results for maize genes, organized for easy navigation and selective access. Note: if pasting commands from this document, please make sure command is on one continuous line in the terminal/shell.

1. Downloading the Dataset

The complete compressed dataset (approximately 255 GB) is available as a single file from Zenodo (**note**, the download can take from 30 minutes to several hours, depending on internet connection).

a. Download Link:

https://zenodo.org/records/17402684

File Name: Maize_ScanFold_Data_Archive.tar.gz

OR

b. Use the zenodo command line tool:

If `pip` is not installed: `$ wget https://bootstrap.pypa.io/get-pip.py`
If `zenodo-get` is not installed: `$ pip install zenodo-get`

Download command:
```
$ zenodo_get 17402684
```

You have two primary options for accessing the data: extracting the entire archive or selectively extracting specific gene folders. Choose based on your available disk space and needs.

2. Option A: Full Extraction (High Disk Space Required)

Select this option if you have over 255 GB of free disk space and want the entire dataset readily available for exploration.

For Linux and macOS:
Open a terminal and execute the following command. Using `pigz` is recommended for faster decompression compared to standard `gzip`.

If `pigz` is not installed:

Linux (Ubuntu/Debian): `$ sudo apt-get install pigz`
macOS (with Homebrew): `$ brew install pigz`

Extraction Command:

```
$ pigz -dc Maize_ScanFold_Data_Archive.tar.gz | tar -xvf -
```

For Windows:
We recommend using the free software 7-Zip for extraction.

Download and install 7-Zip from https://www.7-zip.org/.
Right-click on the Maize_ScanFold_Data_Archive.tar.gz file.
Select “7-Zip” > “Extract Here”.

Data Organization:
Once extracted, the archive creates a main folder named Maize_ScanFold_Data. Inside this folder, the data is organized into subfolders, one for each maize gene (e.g., Zm00001eb190010/). This structure allows easy navigation to specific genes of interest.

3. Option B: Partial Extraction (Advanced, Low Disk Space)

If you only need data for specific genes, avoid decompressing the entire 255 GB archive. The following methods enable efficient extraction of individual subfolders. These are primarily for Linux and macOS users.

Method 1: Streaming Extraction Using pigz and tar
This method streams through the archive and extracts only the specified folder without full decompression.

Step 1: Install Required Tool (pigz)

Linux (Ubuntu/Debian): `$ sudo apt-get install pigz`
macOS (with Homebrew): `$ brew install pigz`

Step 2: Extract Your Gene of Interest

For example, to extract data for the gene Zm00001eb190010:
Create an output directory:

```
$ mkdir Zm00001eb190010_output
```

Run the extraction command:

```
$ pigz -dc Maize_ScanFold_Data_Archive.tar.gz | tar -xvf - -C
Zm00001eb190010_output Maize_ScanFold_Data/Zm00001eb190010/
```

Command Breakdown:
-C Zm00001eb190010_output: Specifies the output directory for extracted files.
/lustre/hdd/LAS/wmoss-lab/masoneis/Maize_ScanFold_Data/Zm00001eb190010/: The full path inside the archive to the desired folder. Replace Zm00001eb190010 with your gene of interest for other extractions.

Method 2: Mounting the Archive Using ratarmount
This tool "mounts" the archive as a read-only filesystem, allowing you to browse and copy files without extraction. It requires Python and pip.

Step 1: Install Required Tool (ratarmount):

```
$ pip install ratarmount
```

Step 2: Create a mount point directory:

$ mkdir mountpoint

```
Step 4:Mount the archive:

$ ratarmount Maize_ScanFold_Data_Archive.tar.gz mountpoint/
```

The first mount may take time to build an index file. Subsequent mounts are fast.
Your terminal will appear to "hang" – this is normal; the mount is active.
Open a new terminal for the next steps

Step 5 : Copy the entire gene folder (e.g., to your home directory):

```
$ cp -r mountpoint/Maize_ScanFold_Data/Zm00001eb190010/
~/my_extracted_gene/
```

When finished, unmount the archive (from the new terminal):

Linux: `$ fusermount -u mountpoint`
macOS: `$ umount mountpoint`
