# Supplementary material for: Transcriptome-wide prediction of heat-sensitive RNA structures in Zea mays
Source: Front Plant Sci. 2025 Nov 26;16:1688991. doi: 10.3389/fpls.2025.1688991 (PMC12689509; doi:10.3389/fpls.2025.1688991)
Supplement: Supplementary file 2 [file DataSheet2.pdf]

Supplementary Material

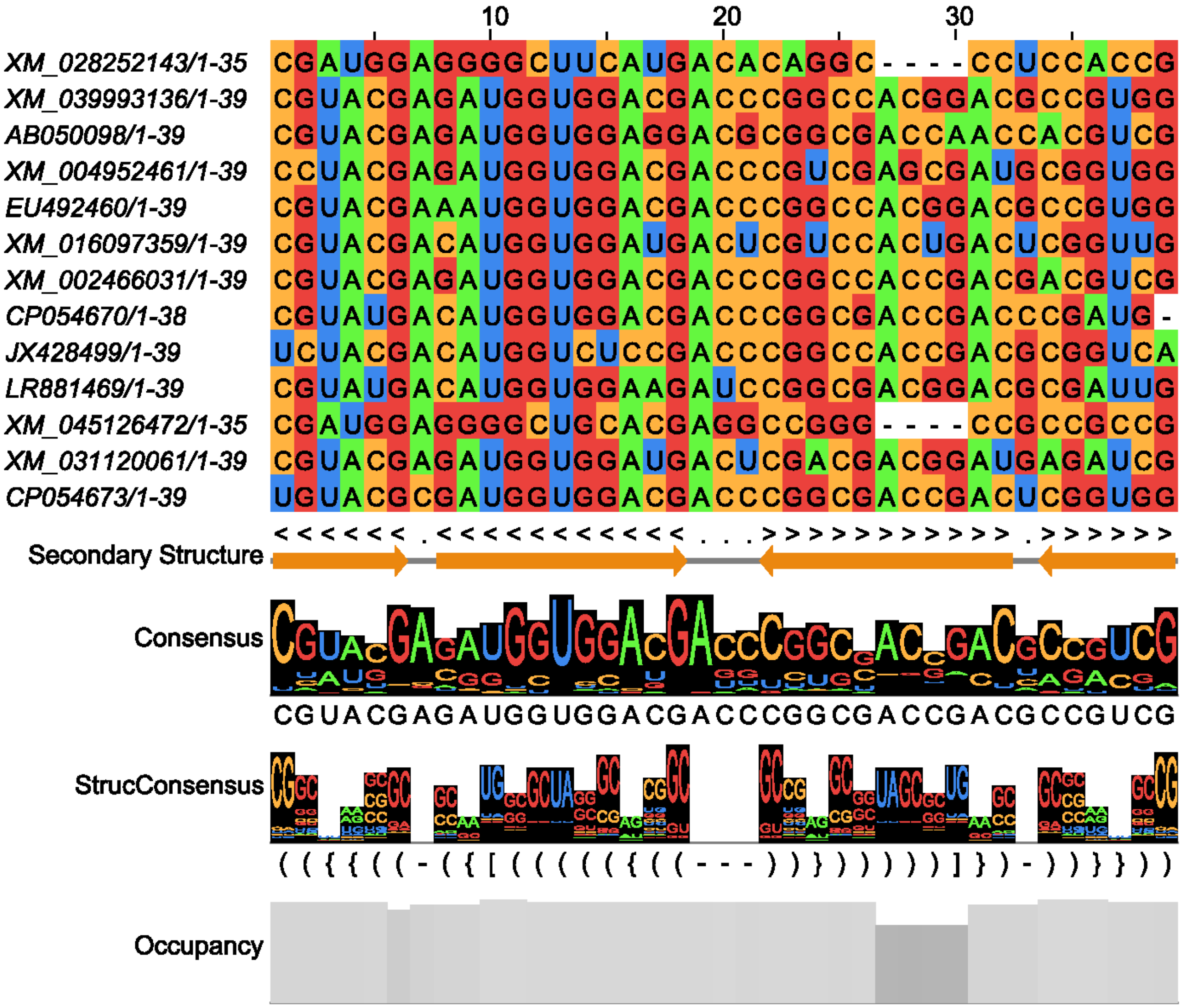

Supplementary Figure S1. Abbreviated stockholm alignment from the Cobretti pipeline of a structured region in *ZmHsf04*. Includes secondary structure, consensus sequence, structure consensus, and occupancy for each nucleotide. Organisms are listed by accession from the BLAST database.

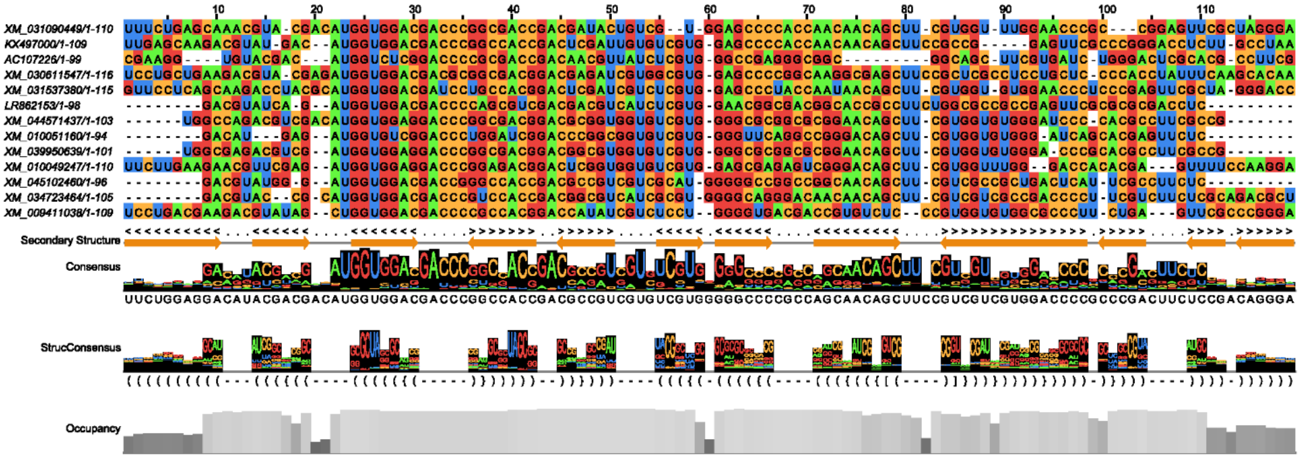

Supplementary Figure S2. Abbreviated stockholm alignment from the Cobretti pipeline of a structured region in *ZmHsf17*. Includes secondary structure, consensus sequence, structure consensus, and occupancy for each nucleotide. Organisms are listed by accession from the BLAST database.

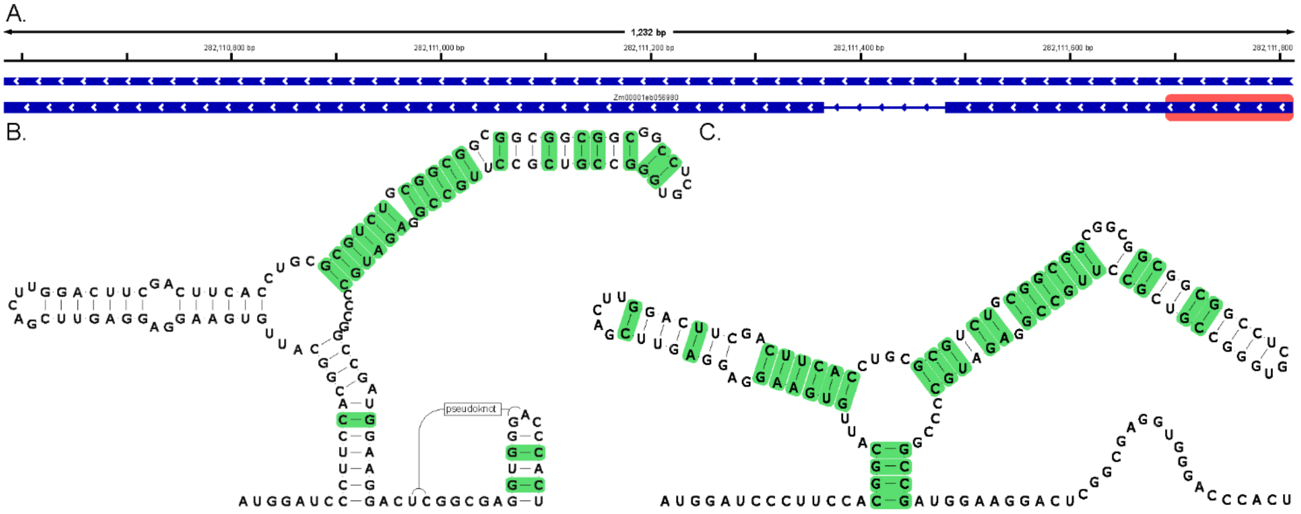

Supplementary Figure S3. Local region data showing a covarying structural motif in its different predicted forms. **(A)** Isoforms for *ZmHsf17* with the local region of interest highlighted in light red. **B)** Knotty predicted structure with covarying pairs shown highlighted in green, and a pseudoknot as marked. **(C)** ScanFold predicted structure with covarying pairs shown highlighted in green.

Supplementary Table S1. Heat shock factor family gene names, gene IDs, average ΔEDs, and chromosome number as denoted in the *Zea mays B73-V5* reference genome.

Gene Name	Gene ID	Average ΔED	Chromosome #
ZmHsf01	Zm00001eb100770	3.408071	2
ZmHsf02	Zm00001eb100410	2.742928	2
ZmHsf03	Zm00001eb160080	3.258603	3
ZmHsf04	Zm00001eb301280	3.584940	7
ZmHsf05	Zm00001eb064860	3.064518	1
ZmHsf06	Zm00001eb242480	3.107025	5
ZmHsf07	Zm00001eb198620	2.236543	4
ZmHsf08	Zm00001eb037600	3.475522	1
ZmHsf09	Zm00001eb399800	2.352485	9
ZmHsf10	Zm00001eb319350	2.701613	7
ZmHsf11	Zm00001eb060670	3.095926	1
ZmHsf12	Zm00001eb384070	3.645868	9
ZmHsf13	Zm00001eb004670	3.701523	1
ZmHsf14	Zm00001eb009170	2.977732	1
ZmHsf15	Zm00001eb293250	3.622844	6
ZmHsf16	Zm00001eb353710	2.616222	8
ZmHsf17	Zm00001eb056980	4.126346	1
ZmHsf18	Zm00001eb239380	2.709545	5
ZmHsf19	Zm00001eb017880	3.478349	1
ZmHsf20	Zm00001eb428800	3.462089	10
ZmHsf21	Zm00001eb314890	3.307526	7
ZmHsf22	Zm00001eb210530	3.175188	5
ZmHsf23	Zm00001eb159390	3.943646	3
ZmHsf24	Zm00001eb047760	2.958283	1
ZmHsf25	Zm00001eb358700	3.535945	8
ZmHsf26	Zm00001eb371000	3.242104	8
ZmHsf27	Zm00001eb328900	3.288808	7
ZmHsf28	Zm00001eb384760	3.380110	9
ZmHsf29	Zm00001eb241300	3.805316	5
ZmHsf30	Zm00001eb314790	3.340172	7
ZmHsf31	Zm00001eb154070	2.539428	3
